# Immunologic response to vaccine challenge in pregnant *PTPN22* R620W carriers and non-carriers

**DOI:** 10.1371/journal.pone.0181338

**Published:** 2017-07-19

**Authors:** Shelly H. Tien, Juliet N. Crabtree, Heather L. Gray, Erik J. Peterson

**Affiliations:** 1 Division of Maternal-Fetal Medicine, Department of Obstetrics, Gynecology, and Women’s Health, University of Minnesota, Minneapolis, Minnesota, United States of America; 2 Center for Immunology and Division of Rheumatic and Autoimmune Diseases, Department of Medicine, University of Minnesota, Minneapolis, Minnesota, United States of America; Imperial College London, UNITED KINGDOM

## Abstract

**Objectives:**

Influenza infection is a significant cause of respiratory morbidity among pregnant women. Seasonal influenza vaccination engages innate immune receptors to promote protective immunity. A coding polymorphism (R620W) in *PTPN2*2 imparts elevated risk for human infection and autoimmune disease, predisposes to diminished innate immune responses, and associates with reduced immunization responses. We sought to quantify the effects of *PTPN22-*R620W on humoral and cell-mediated immune responses to the inactivated influenza vaccine among healthy pregnant women.

**Study Design:**

Immune responses were measured in healthy pregnant R620W carrier (n = 17) and non-carrier (n = 33) women receiving the 2013 quadrivalent inactivated influenza vaccine (Fluzone). Hemagglutination inhibition assays were performed to quantify neutralizing antibodies; functional influenza-reactive CD4 T cells were quantified by flow cytometry, and influenza-specific CD8 T cells were enumerated with MHC Class I tetramers. Antibody seroconversion data were evaluated by Chi-square analysis, and the Mann-Whitney or Wilcoxon signed-rank tests were applied to T cell response data.

**Results:**

*PTPN22* R620W carrier (n = 17) and non-carrier (n = 33) groups did not differ in age, parity, BMI, gestational age at time of vaccine, or history of prior influenza vaccination. After Fluzone exposure, 51.5% of non-carriers met criteria for antibody seroconversion to H1N1 influenza, compared with 23.5% of R620W carriers (p = 0.06). Influenza-reactive CD4 T cells showed modest increase at days 9–15 after vaccination in both R620W carriers and non-carriers (p = 0.02 and p = 0.04, respectively). However, there was no difference in overall response between the two groups (p = 0.6). The vaccine did not result in significant induction of influenza-specific CD8 T cells in either group.

**Conclusions:**

There was no significant difference among healthy pregnant R620W carriers and non-carriers in H1N1 antibody seroconversion rates after influenza vaccination. Studies of larger cohorts will be needed to define the effect of *PTPN22* risk allele carriage on antibody and T cell responses to influenza vaccination during pregnancy.

## Introduction

Pregnancy is a well-recognized risk factor for heightened susceptibility to, and increased severity of, respiratory and other infections. Multiple physiologic adaptions of pregnancy, including a reduced pulmonary functional residual capacity, a 30–50% increase in minute ventilation and tidal volume, mechanical cephalad displacement of the diaphragm, and increased metabolic demands of the fetus, likely contribute to increased morbidity of respiratory infections in pregnancy [1]. Furthermore, hormonal and immunologic variations in pregnancy likely play a crucial role in both infectious disease susceptibility and severity [2].

Especially in pregnant women, seasonal influenza infection remains a significant cause of respiratory morbidity and mortality in the United States [3]. Compared to non-pregnant adults, pregnant women suffer greater influenza-related morbidity, with increased severity of symptoms, more frequent medical visits, and higher rates of hospitalization. In every influenza pandemic since the beginning of the 20th century (1918–1919, 1957–1958, 2009), mortality was disproportionately increased among pregnant women [4–7]. Given the tremendous disease burden reflected in pregnant populations, and observations that influenza vaccination during pregnancy can reduce the risk of influenza infection by 50% [8], both the CDC and the American College of Obstetricians and Gynecologists recommend annual inactivated influenza vaccination for all pregnant women [9,10], typically as soon as possible in pregnancy.

Despite its reported benefits in pregnant women, influenza vaccination is far from a panacea for protection against infection. Recent meta-analysis estimated the overall efficacy of the seasonal inactivated influenza vaccine in adults at only 59% [11]. Seroconversion, defined as a fourfold increase in antibody titers after influenza vaccination, is observed in 41%-78% of healthy non-pregnant subjects [12,13]. The preponderance of the data suggests that pregnant women mount humoral responses to influenza vaccination similar to those observed in the non-pregnant population [14–19]. In one large randomized controlled trial, pregnant women demonstrated incidence of antibody seroconversion (39%-83%) to influenza vaccination comparable to that observed in non-pregnant adults [15]. However, other reports suggest that pregnancy is associated with diminished antibody responses to certain influenza vaccine strains [20]. Since pregnant women suffer greater morbidity associated with active influenza infection, addressing the problem of limited vaccine efficacy is particularly important for the pregnant population.

Immunogenicity of anti-viral vaccination varies greatly in both pregnant and non-pregnant populations [21]. Both host and environmental factors may contribute to vaccine immunogenicity. Host variables known to modulate vaccine immunogenicity include age, presence of autoimmune disease, and use of immunosuppressive medications [22]. Cellular and molecular determinants of vaccine immunogenicity have also recently been defined in animal studies [23]. Innate immune cells of the myeloid lineage respond to influenza vaccination via pattern recognition receptors that signal cellular activation and trigger the adaptive immune response. Toll-like receptor (TLR) signals and type 1 Interferons (IFN-1) expressed by myeloid cells including dendritic cells are necessary for optimal T cell-dependent B cell responses including production of high-affinity neutralizing antibodies.

Knowledge of the molecular underpinnings for myeloid cell TLR signaling upstream of IFN-1 promoters is advancing rapidly. A requirement for Protein tyrosine phosphatase non-receptor type 22 (*PTPN22*) in optimal myeloid cell production of type 1 IFN was recently established [24]. *PTPN22* is widely expressed in hematopoietic tissue and functions as a negative regulator of T cell antigen receptor signaling [25–27]. However, in innate immune cells, *PTPN22* is a positive regulator of TLR3/4/7/9 signals that lead to transactivation of IFN-1. Further, *PTPN22* potentiates dendritic cell activation, supports optimal expansion of virus-specific CD8 T cells, and protects against lethality after viral infection *in vivo* [24].

A single nucleotide polymorphism in the *PTPN22* gene (rs2476601) imparts altered risk for infection and autoimmune disease [28]. Carriage of the *PTPN22* risk allele is associated with increased susceptibility to major autoimmune disorders such as diabetes, thyroid syndromes, rheumatoid arthritis, systemic lupus erythematosus, and myasthenia gravis, as well as with altered host responses to bacterial and fungal infections [28,29]. The *PTPN22* risk allele encodes an amino acid substitution from arginine-620 to tryptophan (R620W), but the mechanism(s) whereby the R620W variant predisposes to disease are not well-understood [27]. *PTPN22* R620W exhibits “loss of function” in TLR signaling and in type 1 IFN production in both animal models and in human peripheral blood cells [24]. These findings suggest that R620W carriage may impair innate immune responses entailing type 1 IFN production, including host response to anti-viral immunization. Our recent study [30] in healthy non-pregnant adults demonstrated decreased expansion of influenza virus-specific CD4 T cells as well as a blunted antibody affinity response following influenza vaccination in R620W variant carriers, demonstrating that the R620W variant may have important implications for vaccine efficacy. The role of R620W in modulating human immune responses to vaccination in the setting of pregnancy has not yet been studied.

The above observations led us to hypothesize that pregnant *PTPN22* R620W carriers would mount dampened T cell and influenza-specific neutralizing antibody responses compared to non-carriers after influenza vaccination. To test this hypothesis, we measured the humoral and cell-mediated immunologic response to the inactivated influenza vaccine among healthy pregnant women who are *PTPN22* R620W carriers and non-carriers.

## Materials and methods

### Subjects

We recruited 114 healthy pregnant women receiving routine care in a University obstetrical clinic from September to November 2013. Exclusion criteria included age < 18, a history of autoimmune disease, and non-English speaking status. *PTPN22* R620W (RW) carrier (variant) subjects (n = 17) and a group of non-carrier (RR) subjects (n = 33) were selected in a 1:2 ratio based on patient availability and ability to complete the study. Participants submitted blood samples pre-influenza vaccine, as well as 9–15 days and 15–30 days post-vaccine. Subjects received the intramuscular Fluzone quadrivalent vaccine (Sanofi Pasteur Inc., 2013) against strains A/California/7/2009(H1N1), A/Texas/50/2012(H3N2), B/Massachusetts/2/2012/Yamagata, and B/Brisbane/60/2008/Victoria [31]. The gestational age of vaccine receipt depended upon time of patient presentation to prenatal care as well as vaccine availability in the clinic. IRB approval (#1210M21901) was obtained through the University of Minnesota IRB Human Subjects Committee, and all subjects provided informed written consent. Variable cellular yields from PBMC isolation procedures determined the listed group sizes for individual T cell assays.

### Genotyping

DNA extraction from whole blood was performed using the DNeasy Blood and Tissue kit (Qiagen). PTPN22 C1858T genotype on all subjects was determined by TaqMan SNP Genotyping (Applied Biosystems). HLA-A genotype was determined through PCR amplification [32], purification of PCR product using QIAquick PCR Purification Kit (Qiagen), and Sanger sequencing. HLA-A type was determined by BLAST of PCR sequence against the IMGT/HLA database.

### Hemagglutination inhibition assay

Whole blood was collected into Serum Separator Tube (SST) Vacutainers (BD) and serum was obtained by centrifugation. Aliquots of serum were mixed with Aprotinin (Sigma) before freezing. Hemagglutination inhibition assays (HAI) were performed using the WHO 2013–2014 Influenza Reagent Kit and Turkey red blood cells in Alsevers (Colorado Serum) according to the manufacturer’s instructions. Antibody seroprotection was defined as a HAI titer of at least 1:40, and seroconversion was defined as a fourfold increase in titer after vaccination [20].

### CD4 T cell assessments

Pre- and post-vaccine whole blood was collected into CPT Vacutainers (BD) and centrifuged to isolate peripheral blood mononuclear cells (PBMC) which were aliquoted and frozen. PBMCs were thawed and washed with prewarmed RPMI 1640 containing 10% FBS and 1% Pen/Strep and rested before stimulating with either 10uL of the 2013–2014 Fluzone vaccine or PMA (50ng/ml) and ionomycin (1ug/mL) as a positive control, in the presence of Brefeldin A as a negative control. Cells were stimulated for 6 hours at 37 degrees Celsius. Cells were stained with live/dead Aqua (Biolegend) and surface antibodies, before permeabilization and intracellular staining. Cells were stained for a panel of antibodies, including CD4, IL-2, IFNγ and TNFα (BD, Biolegend, eBioscience, Tonbo Biosciences). Cells were run on an LSRFortessa (BD) and data were analyzed using FlowJo software (Tree Star). Gates for cytokine production were determined using no stimulation control performed on each sample. Influenza virus-specific responses were determined by subtracting numbers of cytokine-producing cells in no stimulation control samples from the total cytokine-producing CD4 fraction observed in Fluzone-stimulated samples.

### CD8 T cell assessments

We identified influenza-specific CD8 T cells in subjects harboring human leukocyte antigen HLA-A*02 using influenza peptide-containing MHC Class I tetramers of HLA-A*02. PBMCs were thawed and stained with HLA-A*0201 Influenza M1 tetramer for peptide GILFVFTL (Beckman Coulter) [33] along with surface antibodies before permeabilizing and intracellular staining. Antibodies (BD, Biolegend, eBioscience, Invitrogen) against CD38, HLA-DR, Ki-67, and Granzyme B were used. Cells were enumerated by flow cytometry.

### Statistics

We performed power calculations based upon the magnitude of differences between human R620W carriers and non-carriers observed in a previous study of LPS-induced type 1 IFN-dependent gene upregulation in PBMC [24]. To reach a power of 80% to detect a one standard deviation difference at a significance level of 0.05 between carrier and non-carrier groups in humoral and cell-mediated responses to influenza vaccination, we calculated a requirement for at least 16 subjects in each group. Chi-square or Fisher’s exact tests were used to compare categorical variables (antibody seroconversion and seroprotection), and the t test was employed to analyze the geometric mean ratio. The Mann-Whitney test was performed for non-parametric variables of CD4 and CD8 T cell induction, as well as for evaluation of increases in antibody titers. The Wilcoxon signed-rank test was employed for paired non-parametric values. Statistical significance was set at p<0.05. Statistical analysis was performed using SAS 9.3 and GraphPad Prism 6 software.

## Results

Responses to the 2013 quadrivalent inactivated influenza vaccine (Fluzone) were studied in a cohort of pregnant women comprising 17 *PTPN22* R620W carriers (RW) and 33 non-carriers (RR). Genotyped groups showed no differences in demographic or clinical characteristics, including age, parity, BMI, ethnicity, gestational age at time of vaccine, time to post vaccine blood draws, or history of prior influenza vaccination (Table 1).

**Table 1:** Pregnant subject characteristics.

	All subjects (N = 50)	R/R* (N = 33)	R/W* (N = 17)	P value
Values are Mean (SD)				
Maternal age in years	31.1 (4.4)	31.7 (3.8)	29.9 (5.2)	0.2
Gestational age at time of vaccine in weeks	25.5 (9.2)	25.9 (9.1)	24.8 (9.6)	0.7
BMI				
Pre-pregnancy	24.4 (4.9)	24.2 (5.0)	24.3 (4.9)	0.9
At time of vaccine	27.4 (5.6)	27.3 (5.4)	27.5 (6.2)	
Days post vaccine draw				
9–15	12.2 (1.9)	12.4 (2.0)	11.9 (1.9)	0.4
15–30	23.4 (3.9)	23.9 (4.3)	22.3 (2.9)	0.2
Change in days	11.1 (4.3)	11.5 (4.5)	10.4 (3.9)	0.4
Values are N (%)				
Parity				
0	25 (50.0)	16 (48.5)	9 (52.9)	0.8
>0	25 (50.0)	17 (51.5)	8 (47.1)	
Ethnicity				
Caucasian	47 (94.0)	30 (90.9)	17 (100.0)	0.5
Non-Caucasian	3 (6.0)	3 (9.1)	0 (0.0)	
Smoking				
Yes	2 (4.0)	1 (3.03)	1 (5.9)	1.0
No	48 (96.0)	32 (96.9)	16 (94.1)	
HLA-A02				
Yes	23 (46.0)	14 (42.4)	9 (52.9)	0.5
No	27 (54.0)	19 (57.6)	8 (47.1)	
Fetal Number				
Singleton	43 (86.0)	28 (84.8)	15 (88.2)	1.0
Twins	7 (14.0)	5 (15.2)	2 (11.8)	
Prior influenza vaccine?				
Yes	42 (84.0)	24 (84.8)	14 (82.4)	1.0
No/Unknown	8 (16.0)	5 (15.2)	3 (17.6)	

* R/W = R620W variant carriers; R/R = Non-carriers

Data are expressed as mean (SD) for continuous variables (t-test) and N (percentage) for categorical variables (Chi square)

### Humoral responses

**Fig 1:**
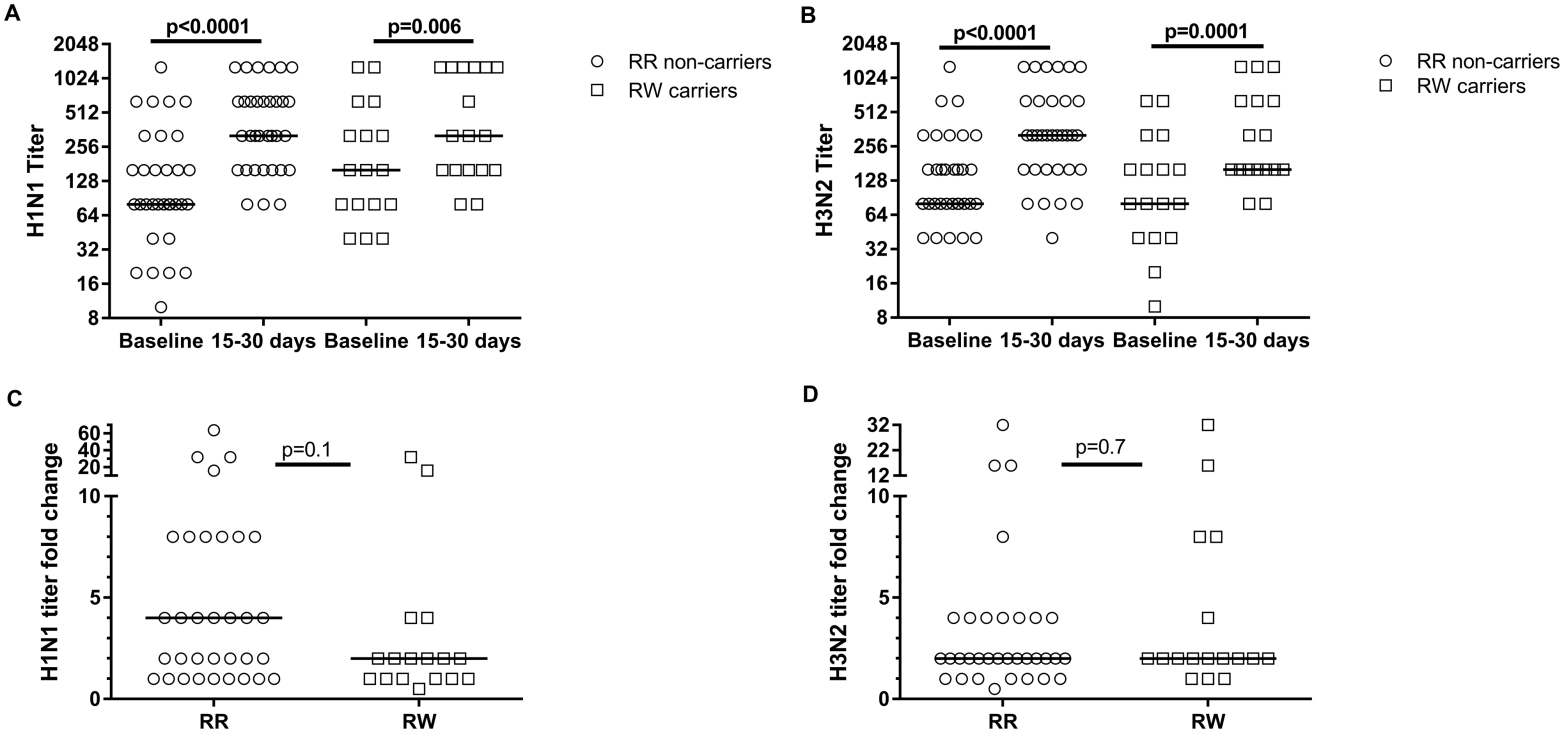
Pregnant PTPN22 R620W carriers (R/W) and non-carriers (R/R) show increases in neutralizing antibody titers after influenza vaccine. Absolute HAI titers for H1N1 (A) and H3N2 (B) were measured at baseline, and at 15–30 days after vaccination. Fold increases in antibody titer to H1N1 (C) and H3N2 (D) at 15–30 days after vaccination are shown. Horizontal lines indicate median values.

Neutralizing serum antibody titers represent the longest-studied correlate of protective anti-influenza immunity [34][3]. We used Hemagglutination inhibition (HAI) assays to assess antibody responses to immunization. Both *PTPN22*-R620W variant carriers and non-carriers demonstrated high rates (84.8–100%) of seroprotection (defined as HAI titer > 1:40) pre-vaccine, and showed complete seroprotection to H1N1 and H3N2 influenza subtypes after vaccination (Table 2). Four of 17 (23.5%) R620W carriers, compared to 17 of 33 (51.5%) non-carriers, demonstrated seroconversion in response to the H1N1 influenza subtype; this difference was not significant (p = 0.06; Table 2). Both genotype groups showed statistically-significant increases in absolute antibody titers after immunization (Fig 1A and 1B). There was no difference in median fold-increase in H1N1 subtype antibody titers between R620W carriers and non-carriers (p = 0.1; Fig 1C) or in the H3N2 subtype (p = 0.7; Fig 1D). There was no significant difference in rates of seroconversion to H3N2 subtype between carriers (29.4%) and non-carriers (33.3%; p = 0.8; Table 2). Notably, overall seroconversion rates for H3N2 were lower than for H1N1, a finding that has been reported previously in both pregnant and non-pregnant women [20].

**Table 2:** There is no difference in seroconversion to H1N1 and H3N2 between carriers and non-carriers.

	R/R (N = 33)	R/W (N = 17)	P***
H1N1			
Pre-vaccine GMT	109.6 (72.6–165.5)	180.8 (99.8–327.6)	0.2
Post-vaccine GMT	378.6 (278.4–514.8)	376.7 (219–647.9)	0.6
Fold Increase, GMR	3.5 (2.3–5.2)	2.1 (1.2–3.6)	0.1
*Seroconversion, N (%)	17 (51.5%)	4 (23.5%)	0.06
**Baseline seroprotection, N (%)	28 (84.8%)	17 (100%)	0.2
H3N2			
Pre-vaccine GMT	129.7 (95.1–176.9)	102.2 (56.6–184.4)	0.8
Post-vaccine GMT	320 (228.6–448)	294.9 (181.4–479.6)	0.9
Fold Increase, GMR	2.5 (1.8–3.4)	2.9 (1.7–4.8)	0.6
*Seroconversion, N (%)	11 (33.3%)	5 (29.4%)	0.8
**Baseline seroprotection, N (%)	33 (100%)	15 (88.2%)	0.1

Data are expressed as mean (95% CI) for continuous variables and N (percentage) for categorical variables. R/W = R620W variant carriers; R/R = Non-carriers. GMR = geometric mean ratio; GMT = geometric mean titer

*Seroconversion is defined as ≥ 4 fold increase in Hemagglutination Inhibition (HAI) titer after vaccination.

**Seroprotection is defined as a HAI titer ≥1:40. All study subjects demonstrated 100% seroprotection to H1N1 and H3N2 after vaccination.

***t test was used to compare GMR and GMT between genotypes. Chi square and Fisher’s exact test were used to compare percentages between genotype seroconversion and seroprotection groups.

### T cell responses

**Fig 2:**
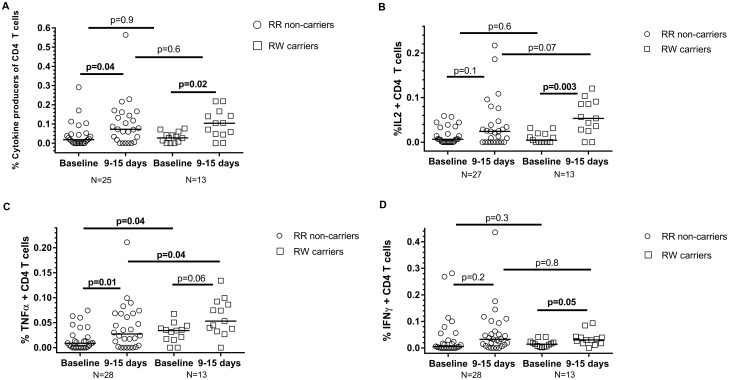
Influenza vaccination induces vaccine-reactive CD4 T cells in pregnant women 9–15 days after vaccination. (A) Frequency, among total CD4 T cells, of CD4 cells producing any of the cytokines IFNγ, IL-2 or TNFα after Fluzone stimulation (6 hours). (B-D) Frequency of CD4 T cells producing IL-2 (B), TNFα (C) or IFNγ (D) among total CD4 T cells after Fluzone stimulation. Horizontal lines indicate median values. P values are determined by Wilcoxon matched-pairs signed rank and Mann-Whitney tests.

CD4 T cells provide “help” that drives B cell activation and antibody production during responses to viral infection and immunization [35]. We evaluated the role of *PTPN22* R620W carriage in CD4 T cell responses to influenza vaccination. To identify influenza-responsive T cells, we stimulated PBMCs with influenza vaccine (Fluzone) *in vitro*. We detected and enumerated cytokine (IFNγ, TNFα, IL-2) -producing CD4 T cells by flow cytometry [36]. Flow cytometry data yield varied for subjects, thus accounting for the variation in available T cell data for statistical analysis. We observed modest, but significantly-increased fractions of influenza-reactive cells among total CD4 T cells at 9–15 days after vaccination, compared to pre-vaccination (Fig 2A). However, we found no significant difference between *PTPN22* R620W variant carriers and non-carriers in influenza-reactive CD4 T cell induction (Fig 2A). We observed statistically-significant increases in TNFα^+^ CD4 T cell fractions after vaccination in non-carriers (Fig 2C). Although higher fractions of TNFα^+^ CD4 T cells were observed in R620W carriers after vaccination, the carriers also displayed higher fractions of pre-vaccine TNFα^+^ CD4 T cells, compared to non-carriers (Fig 2C). No differences between groups were observed in the minimally-increased fractions of vaccination-induced IFNγ^+^ CD4 T cells, which is consistent with the reduced helper T cell type 1 response and dampened production of IFN-γ seen in pregnancy [37] (Fig 2D).

**Fig 3:**
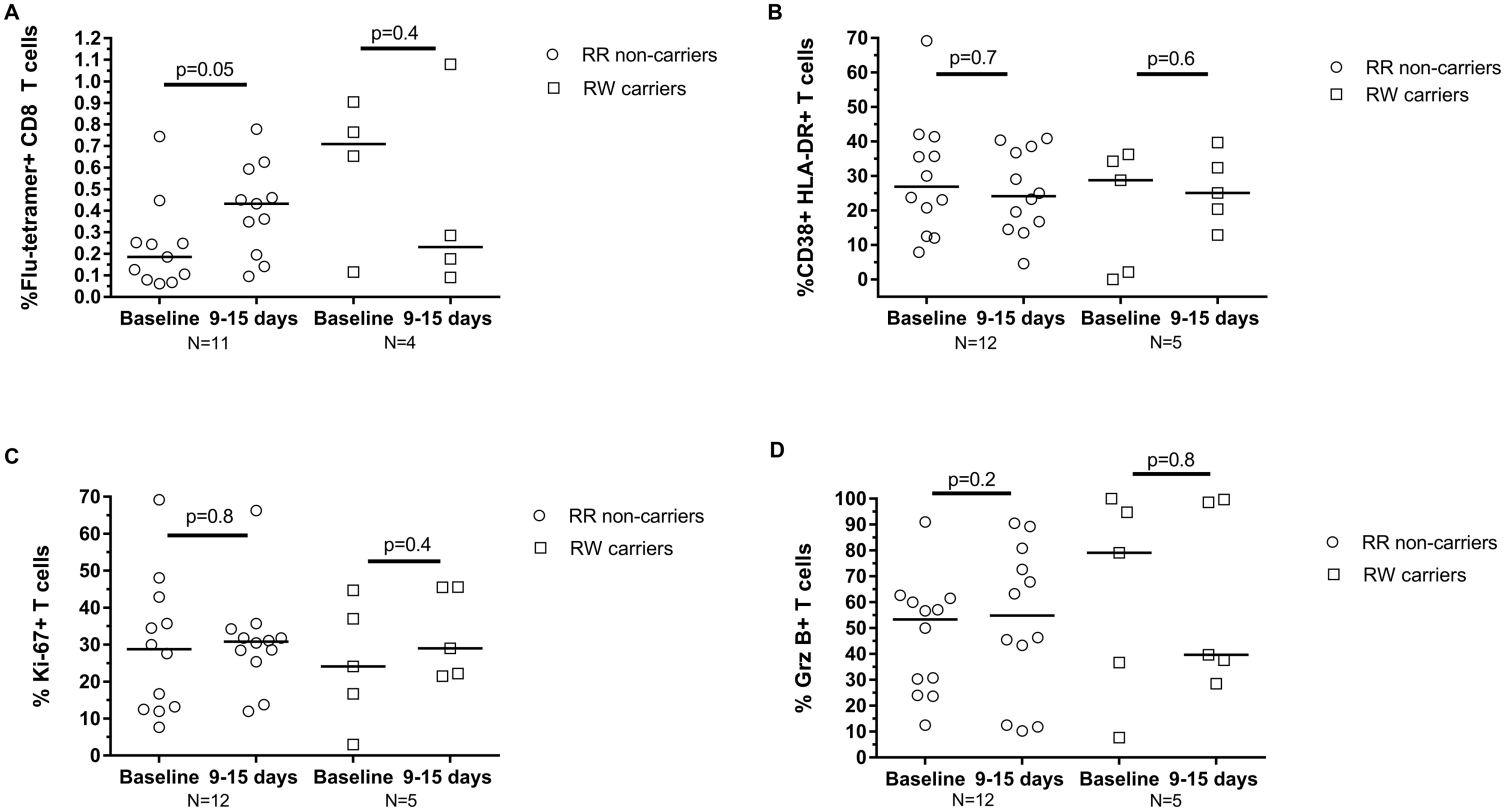
Few influenza-specific CD8 T cells are detectable in PBMC following vaccination of pregnant women. (A) Frequency of influenza-specific tetramer-reactive CD8 T cells among total CD8 T cells. (B) Percentages of CD38^+^ HLA-DR^+^, (C) Ki-67^+^, and (D) Granzyme B^+^ among tetramer-reactive CD8 T cells. Horizontal lines indicate median values. P values are determined by Wilcoxon matched-pairs signed rank test.

Immunization-induced CD8 T cells contribute to viral clearance upon influenza rechallenge [34]. To assess the role of *PTPN22* R620W carriage on CD8 T cell responses to immunization, PBMC from subjects harboring Class I MHC HLA-A*02 were stained with HLA-A2 tetramers containing a peptide from the Influenza M1 protein [38]. Influenza-specific (tetramer-reactive) CD8 T cells were detected by flow cytometry. We found no significant change in influenza-specific CD8 T cell numbers in either *PTPN22* R620W carriers or non-carriers after influenza vaccination (Fig 3A). Moreover, we found no vaccination-associated increase in CD8 T cells staining positive for CD38, Ki-67, or Granzyme B, markers for cell activation and proliferation, and effector function, respectively, in the cohort (Fig 3B–3D).

## Discussion

Our study reports on humoral and cell-mediated influenza vaccine responses in pregnant women carrying a genetic factor that could modulate vaccination responses (*PTPN22* R620W), and characterizes the viral epitope-specific CD8 T cell responses to influenza vaccine in pregnancy. We observed that *PTPN22* R620W variant pregnant carriers exhibited comparable rates of CD4 T cell expansion and seroconversion rates to H1N1 compared to non-carriers. We confirmed that that modest influenza-specific CD4 T cell increases, but not CD8 T cell responses, can be detected in pregnant women following influenza vaccination. However, diminished overall immune responsiveness in pregnant subjects may have reduced our capacity to detect R620W-dependent alteration in the influenza vaccination response.

PTPN22-R620W constitutes a major “risk” allele for human autoimmune and infectious disease that may confer loss-of-function in anti-viral host defense [27]. R620W-associated defects in CD4 T cell response to influenza vaccination recently observed by our group [30] were not reproduced in this study. Among healthy non-pregnant adults, we found that R620W carriage associated with a fold-change expansion of circulating influenza-specific CD4 T cells approximately half that observed in non-carriers [30]. In contrast, among pregnant subjects, we found no difference between R620W carriers and non-carriers in vaccine-induced CD4 T cell expansion. Likely factors influencing lack of congruence between vaccine response results in pregnant and non-pregnant subjects carrying R620W likely include pregnancy-induced blunting of cellular immune responses, and very high rates of pre-existing influenza humoral immunity observed among pregnant subjects.

Pregnancy induces profound immunologic alterations that establish maternal tolerance of the semi-allogeneic fetal “graft”, but also may result in dampening of maternal protective antibody responses to infection or anti-viral vaccination [39][40]. We observed a vaccine-induced fold-increase (2.0) in influenza-specific CD4 T cells among pregnant subjects that was lower than in non-pregnant subjects (2.5 fold)[30], and at the low end of the range of vaccine-induced CD4 changes reported by others for non-pregnant healthy adults [36, 41–42]. These data suggest that generally blunted vaccine-inducible CD4 T cell responses associated with pregnancy may have contributed to our inability to detect a R620W-dependent effect in the cohort.

Specific cytokine-producing T cell subsets induced by vaccine in the pregnant subjects varied. There was a significant increase in non-carriers of TNFα+ CD4 T cells after vaccination, though not in carriers. There was also a significant difference in the baseline levels of TNFα+ CD4 T cells between the two groups, as well as the post vaccination response. Carriers had a significant increase in IL2+ T cells after immunization. For IFNγ CD4 T cells, there was no difference in baseline or response post vaccination between carriers and non-carriers, or within each individual group’s T cell production (p = 0.2 for non-carriers, p = 0.05 for carriers). These data are consistent with observations of skewing towards Th2 in pregnancy and a reduction in Th1 response (IFNγ–secreting T cells) [37]. However, varying T cell yields after flow cytometry precluding inclusion of all subjects makes interpretation of this T cell subset data challenging.

Overall post-vaccination seroconversion rates of 37.5% for H1N1 and 31.4% to H3N2 in the present study are lower than previously reported for pregnant women. Others reported 84–95% seroconversion to H1N1 influenza subtype [15–17, 43], and 69% seroconversion to H3N2 subtype [15,16], in pregnant women after vaccination. The lower overall seroconversion rates that we observed could be influenced by host genetic factors, including *PTPN22* R620W. Another determinant could be the high rate of pre-vaccine seroprotection in the pregnant subjects studied here (88.2–100%). Pre-existing high-affinity antibodies likely facilitate more rapid early clearance of injected immunogens, leading to lower rates of seroconversion to vaccination [44–45]. In our study of non-pregnant adults, these subjects also had high rates of previous vaccination history. For the H1N1 subtype, non-carriers had baseline seroprotection of 88% and carriers had baseline seroprotection of 100%, and for H3N2, 82% and 94%, respectively. Nonetheless, larger studies of pregnant women with lower rates of previous vaccination or influenza infection will be required to ascertain a role for R620W.

We employed MHC-I tetramers to study influenza-specific CD8 T cell responses [33]. Our results confirm previous findings that vaccination-induced CD8 T cell alterations are not robust, regardless of pregnancy status [46,47,33,30]. We found CD38, Ki-67, and Granzyme B expression in virus-specific CD8 T cells to be comparable among pregnant vaccine recipients, and similarly-aged non-pregnant subjects [30]. Pregnancy is not associated with CD8 senescence or functional exhaustion that associates with increased influenza infection and morbidity in vulnerable populations such as the elderly [48]. More informative studies of how genetic (e.g. PTPN22-R620W) or environmental (e.g. pregnancy) factors influence CD8 influenza vaccine responses would likely require sampling of tissues, such as lung and secondary lymphoid organs, where these cells develop and exert protective effects.

Limitations of our study include a relatively small sample size, and a high rate of previous exposure to influenza antigens among study subjects. Thus, the high rates of prior influenza immunization (84% overall) and pre-vaccine seroprotection in our cohort may have decreased our ability to detect R620W-associated response differences to the study vaccine. Coupled with the fact that all subjects were adults with likely prior childhood exposure to influenza infection, it is likely that nearly all had been primed by seasonal influenza A virus before vaccination for this study. In addition, varying yields of our T cell data by flow cytometry limited the presentation of T cell response from all subjects, and may have influenced the statistical significance in data analysis. Furthermore, CD8 T cells were only quantified in subjects displaying HLA-A*02 antigen, and the very low yield of CD 8 T cells is challenging to interpret.

In conclusion, we report on the humoral and cell-mediated responses to the influenza vaccine among healthy pregnant women carrying a genetic variant with putative altered function in the innate host response to infection. Larger studies are needed to better elucidate interactions between pregnancy, host genetic factors such as *PTPN22*-R620W, and humoral and cell-mediated immunologic responses to vaccination.

## Supporting information

S1 File CD4 revised data. (XLSX)

S2 File T cell FlowJo results. (XLSX)

S3 File CD4 T cell pre and post frequency. (XLSX)
